# Soluble egg antigen of *Schistosoma japonicum* induces pyroptosis in hepatic stellate cells by modulating ROS production

**DOI:** 10.1186/s13071-019-3729-8

**Published:** 2019-10-14

**Authors:** De-Long Kong, Fan-Yun Kong, Xiang-Ye Liu, Chao Yan, Jie Cui, Ren-Xian Tang, Kui-Yang Zheng

**Affiliations:** 1 Jiangsu Key Laboratory of Immunity and Metabolism, Department of Pathogenic Biology and Immunology, Laboratory of Infection and Immunity, Xuzhou Medical University, Xuzhou, 221004 Jiangsu People’s Republic of China; 2 Department of Physiology, Xuzhou Medical University, Xuzhou, 221004 Jiangsu People’s Republic of China

**Keywords:** Schistosomiasis, Liver fibrosis, Pyroptosis, ROS

## Abstract

**Background:**

Inflammation-induced dysfunction of hepatic stellate cells (HSCs) is involved in schistosomiasis-associated liver fibrosis, and soluble egg antigen (SEA) is a crucial pathogen-associated molecular pattern associated with liver injury in schistosomiasis. In addition, numerous studies have shown that caspase-1-mediated pyroptosis participates in the development of multiple inflammation-related diseases. However, whether pyroptotic cell death of HSCs is involved in SEA-mediated liver damage is not well understood.

**Methods:**

Primary cultured HSCs and *Schistosoma japonicum*-infected mouse liver tissue were analysed for histological changes and caspase-1 activation, and the role of pyroptosis in the mechanisms underlying SEA-induced HSC death was investigated. Accumulation of reactive oxygen species (ROS) in infected livers and SEA-stimulated HSCs was measured by flow cytometry and immunofluorescence.

**Results:**

Caspase-1 activity was elevated in both liver tissues and HSCs of *S. japonicum*-infected mice. Furthermore, SEA stimulation increased the proportion of pyroptotic HSCs, as shown by lactate dehydrogenase (LDH) release assays and by flow cytometric analysis of propidium iodide (PI) and caspase-1 double staining in cells. In addition, ROS generation was elevated in infected liver tissues and SEA-stimulated HSCs, and ROS inhibition downregulated SEA-induced caspase-1 activation and pyroptosis in HSCs.

**Conclusions:**

Our present study demonstrates that pyroptotic cell death in HSCs induced by SEA via ROS-mediated caspase-1 activation may serve as a significant mechanism to initiate the inflammatory response and thereby exacerbate liver injury during *S. japonicum* infection.

## Background

Schistosomiasis is a critical public health problem worldwide. Currently, approximately 260 million people are still suffering from *Schistosoma* infection with different pathological characteristics [1]. Six important species of *Schistosoma* (*S. mansoni*, *S. japonicum*, *S. haematobium*, *S. mekongi*, *S. malayensis* and *S. intercalatum*) can infect humans. *Schistosoma japonicum* is the main epidemic species in China and can cause pathological damage to the hepatic immune system. When a large number of *S. japonicum* eggs are deposited in the liver, soluble egg antigen (SEA) is continuously released into liver tissues. The released SEA can cause egg granuloma formation and fibrogenesis, upon which inflammatory and immune cells are further recruited to the lesion region, leading to a persistent inflammatory response. The inflammation induced by SEA worsens liver fibrosis and constitutes the pathogenic basis of schistosomiasis [2, 3].

It is well known that the functional change in hepatic stellate cells (HSCs) is a key event in the process of liver fibrosis. HSCs can gather around *S. japonicum* egg granulomas during the development of liver pathological damage induced by *S. japonicum* infection [4]. Accordingly, sustained antigenic stimulation in egg granulomas may have a significant impact on HSCs. In addition, *S. japonicum* SEA is capable of triggering the expression of alpha-smooth muscle actin (α-SMA, a marker of activated HSCs) by cultured HSCs [5]. Additionally, SEA stimulation can induce mice peritoneal macrophages to secrete transforming growth factor beta-1 (TGF-β1) and induce HSCs to proliferate and secrete collagen I and collagen III, promoting damage to infected liver tissues [6]. Furthermore, Duan et al. [7] reported that SEA could induce senescence in activated HSC that mediated the clearance of activated HSCs. Therefore, further studies should be performed to clarify the exact effect of SEA on HSCs in schistosomiasis.

Pyroptosis is a pro-inflammatory form of programmed cell death that is mainly induced by the activation of caspase-1 [8, 9]. Pyroptosis is characterized by features of both apoptosis and necrosis and is distinct from other forms of cell death [10]. During pyroptotic cell death, the cell loses membrane integrity, lyses and then releases lactate dehydrogenase (LDH), which is normally maintained within the cell cytosol [11]. Caspase-1-dependent pyroptosis was first reported in mouse macrophages infected with the Gram-negative bacteria *Shigella flexneri* [12]. As a protease, caspase-1 can process the inactive precursors of interleukin-1 beta (IL-1β) into mature inflammatory cytokines [13]. In recent years, multiple studies have confirmed that caspase-1-mediated pyroptosis is involved in infectious diseases, nervous system-related diseases and other inflammatory processes [14–16]. However, the role of pyroptosis in SEA-induced functional changes in HSCs remains unclear.

Inflammation-induced persistent liver injury is one of the important pathological characteristics of schistosomiasis-associated hepatic fibrosis. Previous studies have shown that reactive oxygen species (ROS), the products of oxidative stress, are generated during hepatic fibrosis and that increases in ROS can further promote inflammation and fibrosis [17, 18]. In particular, excessive ROS produced during liver injury associated with *S. mansoni* infection can induce hepatic stress and aggravate hepatic fibrosis [19, 20]. ROS has also been reported to be involved both in pyroptosis induced by cadmium in human umbilical vein endothelial cells and in caspase-1 activation in macrophages during the early stages of leishmaniasis [21]. Nevertheless, whether pyroptosis mediated by caspase-1 is involved in SEA-induced HSC death remains unclear. In addition, whether pyroptosis is related to ROS overproduction during *S. japonicum* infection remains to be clarified. The present study demonstrates that ROS-mediated pyroptosis plays an important role in *S. japonicum* infection related liver injury and SEA-induced HSC death. These findings expand the current understanding of SEA-induced HSC dysfunction and provide new insights to clarify the pathogenic mechanism of schistosomiasis.

## Methods

### Parasites and animals

Female C57BL/6 mice (aged 5–6 weeks, 22 ± 2 g) were purchased from the Comparative Medicine Center of Yangzhou University, Yangzhou, China. The mice were given a standard diet and water and housed in an animal room under specific pathogen-free conditions at 22–25 °C. Forty mice were percutaneously infected with 14 ± 2 *S. japonicum* cercariae that were obtained from infected *Oncomelania hupensis* snails (purchased from the Jiangsu Institute of Parasitic Disease, Wuxi, China). The mice were sacrificed at 3 weeks (early stage), 6 weeks (acute stage), or 12 weeks (chronic stage) post infection (p.i.), and twenty uninfected mice were used as normal controls. Every effort was made to minimize suffering.

### Preparation of SEA

SEA was prepared based on methods previously described [22, 23]. Briefly, freeze-dried *S. japonicum* eggs were mixed with an appropriate volume of sterile PBS (0.01 mol/l, pH 7.2). After being freeze-thawed several times, the mixture was centrifuged at 4 °C and 15000×*g* for 30 min. Finally, the supernatant was sterilized with a 0.22 μm degerming filter, and the protein concentration of SEA was determined with a bicinchoninic acid (BCA) Protein Assay Kit (Bio-Rad, Hercules, USA).

### Preparation and purification of HSCs

HSCs were isolated following methods previously described [24]. In brief, liver was perfused *in situ* with sterile perfusion buffer containing EGTA, followed by perfusion with buffer containing 0.04% type IV collagenase (Invitrogen, Carlsbad, USA) and 0.05% pronase (Roche, Basel, Switzerland) at 37 °C for 8 min. Then, the liver was further digested with perfusion buffer containing 0.08% type IV collagenase, 0.05% pronase and 10 U/ml DNase I (Sigma-Aldrich, St. Louis, USA) at 37 °C in a shaking bath for 20 min. The dispersed cell suspensions were filtered through a 70 μm cell strainer and centrifuged at 4 °C and 580×*g* for 10 min. OptiPrep (10%; Axis-Shield, Dundee, UK) was used for density gradient centrifugation to separate HSCs. Finally, HSCs were purified by using a magnetic-activated cell sorting (MACS) with CD45 microbeads to remove CD45^−^ cells according to the manufacturer’s instructions (Miltenyi Biotec, San Diego, USA). For SEA stimulation, HSCs were seeded onto well plates and then treated with different concentrations of SEA or vehicle. After 24 h, the HSCs were collected as described below. For experiments concerning ROS, HSCs were pre-treated for 30 min with 20 μM N-acetyl cysteine (NAC, ROS inhibitor) before SEA stimulation.

### Immunohistochemistry and quantification

Liver tissue from different groups was fixed with 4% paraformaldehyde, embedded in paraffin, and then sectioned at 4 μm for staining with haematoxylin and eosin (H&E) or Massonʼs trichrome stain. Some paraffin sections of liver tissues were stained for caspase-1 using the primary antibody anti-caspase-1 (Abcam, Cambridge, UK) and horseradish peroxidase-conjugated (HRP) secondary antibody (anti-rabbit IgG). The extent of granuloma formation and hepatic fibrosis and the staining results for caspase-1 were ultimately observed by using light microscopy (Olympus, Tokyo, Japan), and the images were analysed with Image-Pro Plus software (Bio-Rad).

### Western blot analysis

Cells were lysed in lysis buffer with Protease Inhibitor Cocktail (Roche), and the protein concentrations were quantified using a BCA Protein Assay Kit (Bio-Rad). The samples were loaded onto 12% SDS-PA gels and transferred to PVDF membranes (Merck Millipore, Burlington, USA) after electrophoresis. Then, the membranes were blocked with 5% non-fat dry milk containing 0.05% Tween 20 for 2 h and incubated at 4 °C overnight with the appropriate primary antibodies, including antibodies against caspase-1 (Abcam, 1:1000) and β-actin (Abcam, 1:1000). Immunoreactivity was detected with HRP-conjugated secondary antibodies (Abcam, 1: 5000) followed by chemiluminescent substrate development. All samples were analysed in parallel with four replicates. The expression of caspase-1 protein in each sample was normalized to that of β-actin and analysed by using Quantity One software (Bio-Rad).

### Quantitative real-time PCR (qPCR)

Total RNA was isolated from liver tissues or cultured HSCs by using TRIzol Reagent (Invitrogen) according to the manufacturer’s protocol. After reverse transcription of the RNA into cDNA using a HiFi-MMLV cDNA First-Strand Synthesis Kit (Thermo, Shanghai, China), gene expression was analysed by using qPCR with GoTaq qPCR Master Mix (Invitrogen). GAPDH was amplified in parallel as an internal control. The primer sequences were as follows: caspase-1 (F: 5ʹ-ATC TTT CTC CGA GGG TTG G-3ʹ, R: 5ʹAAG TCT TGT GCT CTG GGC AG-3ʹ); GAPDH (F: 5ʹ-TGC ACC ACC AAC TGC TTA GC-3ʹ, R: 5ʹ-GGC ATG GAC TGT GGT CAT GAG-3ʹ).

### Immunofluorescence

Immunofluorescence was performed to detect the expression of caspase-1 and the formation of ROS in primary HSCs. Briefly, the isolated and cultured HSCs were fixed with 4% paraformaldehyde for 30 min, permeabilized with 0.2% Triton X-100 for 15 min, and then blocked with 2% BSA for 30 min. Subsequently, the cells were incubated with anti-caspase-1 and anti-α-SMA antibodies at 4 °C overnight and treated with DyLight 549 goat anti-rabbit IgG and DyLight 488 goat anti-rat IgG secondary antibodies (Invitrogen) in the dark for 1 h. The cells were imaged under a laser scanning confocal microscope (LSCM, Olympus). A ROS assay kit (Abcam) was used to detect the accumulation of ROS in target cells. In brief, the HSCs were loaded with DCFH-DA (20 μM) in serum-free medium in the dark at 37 °C for 20 min and then washed with PBS three times. The results were examined by confocal microscopy.

### Cell viability assay

Cell viability was determined using a Cell Counting Kit-8 (CCK-8) (Dojindo, Kumamoto, Japan). Briefly, HSCs were cultured in 96-well plates to reach the desired confluence. The cells were treated with different concentrations of SEA (0, 25, 50 and 100 μg/ml) for 24 h and then incubated with CCK-8 at 37 °C for 2 h. Finally, the absorbance was measured at 450 nm by using a microplate reader (Tecan, Männedorf, Switzerland).

### LDH release detection

Following treatment of HSCs with different concentrations of SEA, the supernatant was collected, and the release of LDH into supernatant was measured using a commercially available kit (Solarbio, Beijing, China). LDH concentration was quantified by measuring the absorbance at 490 nm using a microplate reader.

### Measurement of ROS in liver tissues

Frozen sections of fresh liver tissues were incubated in dihydroethidium (DHE) diluted with PBS for 30 min at 4 °C in the dark. Microscopy was used to observe and capture images of the dyed tissue sections.

### Flow cytometry

To assess pyroptosis in HSCs, caspase-1 activation was quantified with a FAM-FLICA Caspase-1 Assay Kit (ImmunoChemistry Technologies, Bloomington, USA) following the manufacturer’s instructions. Briefly, after stimulation with SEA for 24 h, the cells were harvested and incubated with caspase-1 detection probe for 1 h in the dark. After the unbound FLICA reagent was removed with washing buffer, the cells were stained with propidium iodide (PI) for 20 min and then analysed with flow cytometry (BD FACSCalibur, Franklin Lakes, USA). Pyroptotic cells were defined as cells that were double positive for activated caspase-1 and PI. For ROS detection, HSCs were cultured on well plates, loaded with DCFH-DA (20 μM) in serum-free medium in the dark for 30 min, and washed three times with wash buffer. The mean fluorescence intensity of intracellular ROS was examined with a FACSCalibur flow cytometer, and the results were analysed with FlowJo software (TreeStar, Ashland, USA)

### Statistical analysis

Student’s t-test and one-way ANOVA were used to compare the data between two groups or among more than two groups, respectively. *P* < 0.05 was considered statistically significant; statistical significance is expressed as follows: *P < 0.05, **P < 0.01, and ***P < 0.001. All statistical analyses were performed with GraphPad Prism software 5.0 (GraphPad Software Inc., San Diego, USA).

## Results

### Caspase-1 activation is associated with liver inflammation and fibrosis in a mouse model of schistosomiasis

**Fig. 1:**
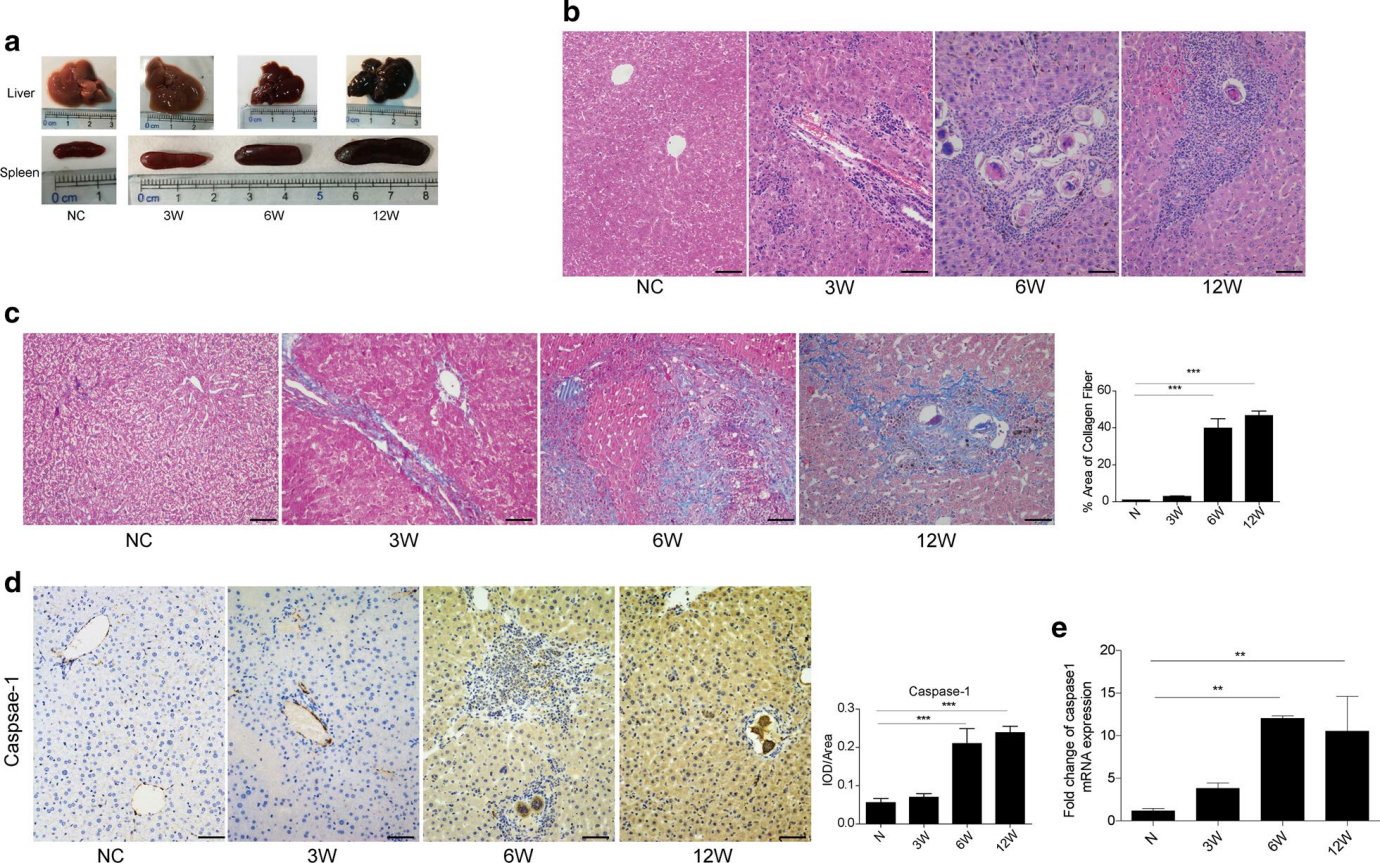
Pathological changes and caspase-1 expression in *S. japonicum*-infected liver tissues. **a** Gross examination of the liver and spleen from normal and *S. japonicum*-infected mice at 3, 6 and 12 weeks post-infection. **b** Representative images of paraffin-embedded liver sections stained with haematoxylin and eosin. *Scale-bars*: 100 μm. **c** Representative images of paraffin-embedded liver sections from immunohistochemical analysis of collagen deposition with Masson’s trichrome staining. The data are presented as the percent positive area. *Scale-bars*: 100 μm. **d** Protein expression of caspase-1 in liver tissues as determined by immunohistochemical analysis under a light microscope and quantitative analysis of positive staining based on the integrated optical density (IOD)/area with Image-Pro Plus software. *Scale-bars*: 100 μm. **e** Relative mRNA expression of caspase-1 determined by qPCR in liver tissues from mice infected with *S. japonicum*. *Abbreviations*: N, livers from naïve mice; 3W, livers from mice infected with *S. japonicum* for 3 weeks; 6W, livers from mice infected with *S. japonicum* for 6 weeks; 12W, livers from mice infected with *S. japonicum* for 12 weeks. The data are presented as the mean ± SEM from five *S. japonicum*-infected mice and four normal control mice at each time point. **P < 0.01, ***P < 0.001

Liver and spleen samples were harvested at 3, 6 and 12 weeks p.i. from mice infected with *S. japonicum*. The results show that we successfully established a mouse model of *S. japonicum* infection in which hepatic injury and spleen enlargement could be observed (Fig. 1a). To evaluate the pathological features in liver tissues at different stages of *S. japonicum* infection, H&E and Masson’s trichrome staining were performed. Our results showed that there were no visible eggs in the liver tissues at 3 weeks p.i. However, typical egg granulomas were visible in the acute (6 weeks p.i.) and chronic (12 weeks p.i.) stages. Meanwhile, a number of infiltrating inflammatory cells were visible at the peripheries of the egg granulomas, and considerable amounts of collagen had accumulated in the damaged areas of the liver, but these effects were not observed in the normal control mice (Fig. 1b, c). To investigate the relationship between liver fibrosis and caspase-1 activation during *S. japonicum* infection, dynamic changes in caspase-1 protein and transcription levels were analysed by using immunohistochemistry and qPCR, respectively. Our results showed that compared to that in non-infected mice, the expression of caspase-1 in infected mice was significantly upregulated at 6 weeks p.i. and persistently increased until the chronic stage at 12 weeks p.i. both protein (ANOVA: *F*_(3, 8)_ = 17.36, *P* < 0.0001) (Fig. 1d) and mRNA levels (ANOVA: *F*_(3, 12)_ = 8.29, *P* = 0.0025) (Fig. 1e). Taken together, our results indicated that liver inflammation and fibrosis were gradually aggravated in infected livers and that caspase-1 activation was involved in these processes caused by *S. japonicum* infection.

### Caspase-1 expression was increased in HSCs isolated from mice in the acute stage of *S. japonicum* infection

**Fig. 2:**
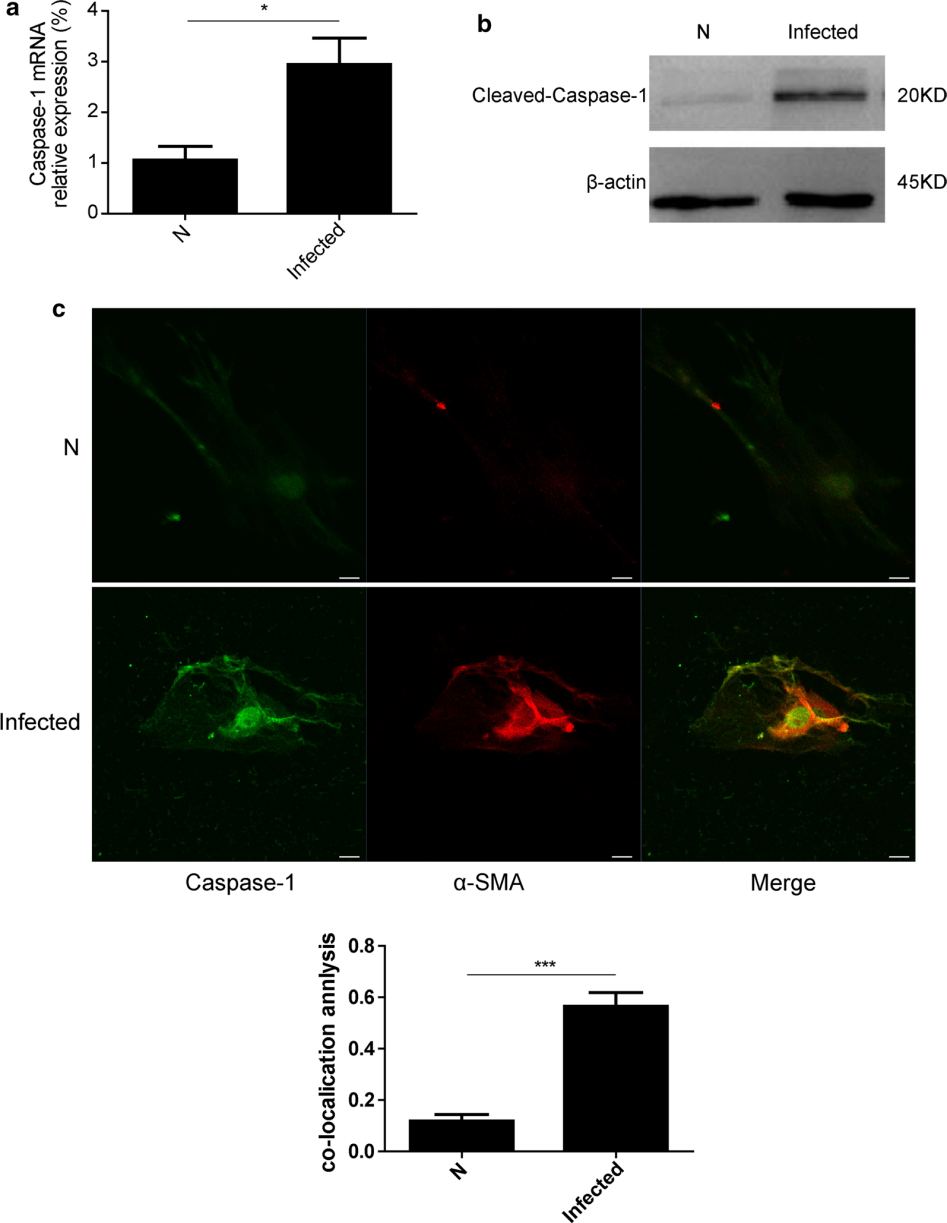
Caspase-1 activation in *S. japonicum*-infected HSCs. **a** The expression of caspase-1 in HSCs isolated from mice 6 weeks post-infection with *S. japonicum* infection was evaluated by qPCR. **b** The expression of caspase-1 protein in HSCs isolated from mice 6 weeks post-infection with *S. japonicum* infection was assayed by Western blot analysis. **c** Representative confocal fluorescence images of the localization of caspase-1 with α-SMA in HSCs isolated from mice 6 weeks post *S. japonicum*-infection and the results of co-localization efficiency analysis (*n* = 5). Scale bar, 20 μm. *P < 0.05, ***P < 0.001

HSCs play the most important role in the pathogenesis of liver fibrosis. To observe changes in caspase-1 in HSCs, we detected the expression of caspase-1 in HSCs derived from livers that were in the initial stage of fibrosis at 6 weeks p.i. The results showed that both the mRNA (t-test: *t*_(6)_ = 3.211, *P* = 0.014) and protein levels of caspase-1 were markedly higher in HSCs from infected mice at 6 weeks p.i. than in HSCs from non-infected mice (Fig. 2a, b). Furthermore, caspase-1 co-localized with the HSC marker α-SMA (t-test; *t*_(6)_ = 15.208, *P* < 0.0001) (Fig. 2c), suggesting that caspase-1 activation occurred in activated HSCs and was involved in schistosomiasis-associated liver fibrosis.

### SEA-induced pyroptosis in HSCs

**Fig. 3:**
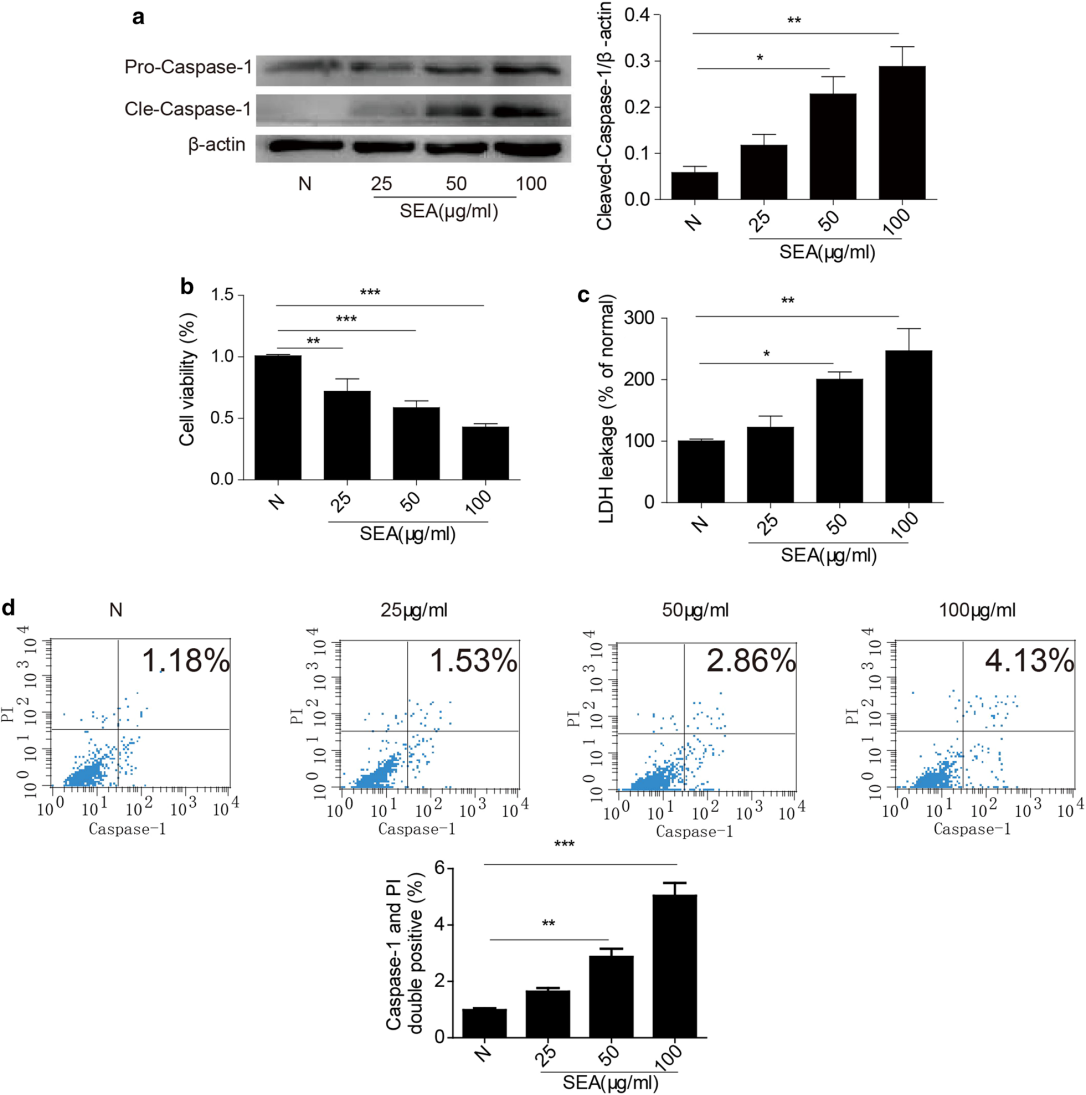
Effect of SEA on pyroptosis in HSCs. **a** The expression levels of pro-caspase-1 and activated caspase-1 proteins in HSCs treated with different concentrations of SEA were determined by Western blot analysis. **b** Dose-dependent effects of SEA on the viability of HSCs. **c** The LDH activity in supernatant was analysed with an LDH cytotoxicity assay kit. **d** Double positivity for caspase-1 and nuclei (stained with PI) in SEA-stimulated HSCs was detected by flow cytometry, and the proportions of double-positive cells determined by statistical analysis are presented in the bar graph. N, primary HSCs isolated from normal mice; 25, 50 and 100, primary cultured HSCs exposed to 25 μg/ml, 50 μg/ml and 100 μg/ml SEA for 24 h, respectively. The data are presented as the mean ± SEM (*n* = 4). *P < 0.05, **P < 0.01, ***P < 0.001

To analyse the effect of SEA on the expression of caspase-1 in HSCs, cells isolated from normal mice were treated with SEA at doses of 25, 50 and 100 μg/ml for 24 h. Western blot analysis showed that the expression of caspase-1 in HSCs was markedly augmented by SEA treatment in a dose-dependent manner (ANOVA: 50 μg/ml: *F*_(3, 12)_ = 10.42, *P* = 0.0219; 100 μg/ml: *F*_(3, 12)_ = 10.42, *P* = 0.0014) (Fig. 3a). Cell viability assays also showed that SEA could inhibit the survival of HSCs in a dose-dependent manner (ANOVA: 25 μg/ml: *F*_(3, 12)_ = 19.41, *P* = 0.0029; 50, 100 μg/ml: *F*_(3, 12)_ = 19.41, *P* < 0.0001) (Fig. 3b). Furthermore, pyroptotic cell death was assessed based on LDH release and double-positive staining for activated caspase-1 and nuclei (stained with PI). Our results showed that SEA (50 and 100 μg/ml) increased LDH release from HSCs (ANOVA: 50 μg/ml: *F*_(3, 12)_ = 12.09, *P* = 0.0239; 100 μg/ml: *F*_(3, 12)_ = 12.09, *P* = 0.0051) (Fig. 3c). We also found that caspase-1 inhibition improved the viability of HSCs (Additional file 1: Figure S1) and reduced the release of LDH (Additional file 2: Figure S2) compared with the SEA group. To further determine whether the death of HSCs induced by SEA was a result of pyroptosis, activated caspase-1 and nuclei (PI staining) were detected using flow cytometry. As shown in Fig. 3d, the proportion of double-positive HSCs was increased in the 50 and 100 μg/ml SEA treatment groups (ANOVA: 50 μg/ml: *F*_(3, 12)_ = 21.31, *P* = 0.0021; 100 μg/ml: *F*_(3, 12)_ = 21.31, *P* = 0.0003), but there was no significant difference in the 25 μg/ml SEA treatment group compared to the control group (ANOVA: *F*_(3, 12)_ = 21.31, *P* = 0.0627). Taken together, our results confirmed that SEA caused pyroptotic cell death in HSCs in a dose-dependent manner.

### ROS generation was increased in both *S. japonicum-*infected liver tissues and SEA-stimulated HSCs

**Fig. 4:**
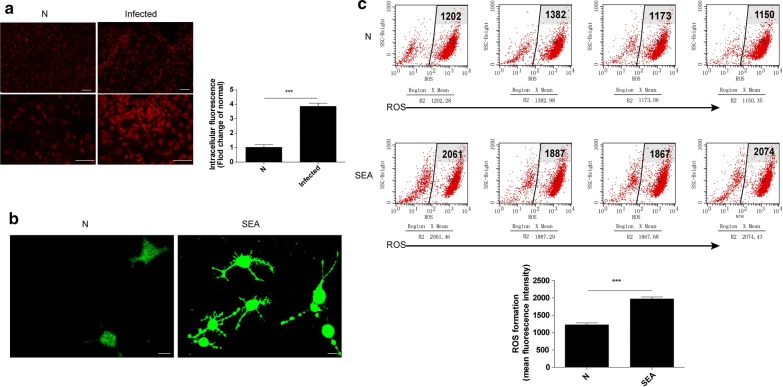
ROS generation was elevated in *S. japonicum*-infected liver tissues and SEA-stimulated HSCs. **a** Representative fluorescence images and statistical analysis results of ROS generation probed by dihydroethidium (DHE) in liver tissue from normal mice and mice at 6 weeks post-infection. *Scale-bars*: 100 μm. **b** Primary cultured HSCs were not stimulated or were stimulated with 50 μg/ml SEA for 24 h, and representative fluorescence images of ROS generation probed by DCFH-DA were obtained under a confocal microscope. *Scale-bars*: 20 μm. **c** The mean fluorescence intensity (MFI) of intracellular ROS was examined and analysed by flow cytometry in HSCs not treated or treated with 50 μg/ml SEA for 24 h (*n* = 4). ***P < 0.001

ROS generation has been demonstrated to be a critical factor for caspase-1 activation. To explore the mechanism underlying caspase-1 activation by *S. japonicum* infection, ROS levels were analysed in *S. japonicum*-infected mouse livers and SEA-treated HSCs by using immunofluorescence. Our results show that ROS levels were significantly increased in the liver tissues of infected mice (t-test: *t*_(6)_ = 10.218, *P* < 0.0001) (Fig. 4a), and SEA treatment promoted intracellular ROS generation in HSCs (Fig. 4b). In addition, the flow cytometry results also showed that the ROS-related mean intracellular fluorescence intensity was clearly higher in SEA-stimulated HSCs than in non-stimulated HSCs (t-test: *t*_(6)_ = 9.756, *P* < 0.0001) (Fig. 4c).

### ROS mediate SEA-induced caspase-1 activation and pyroptosis in HSCs

**Fig. 5:**
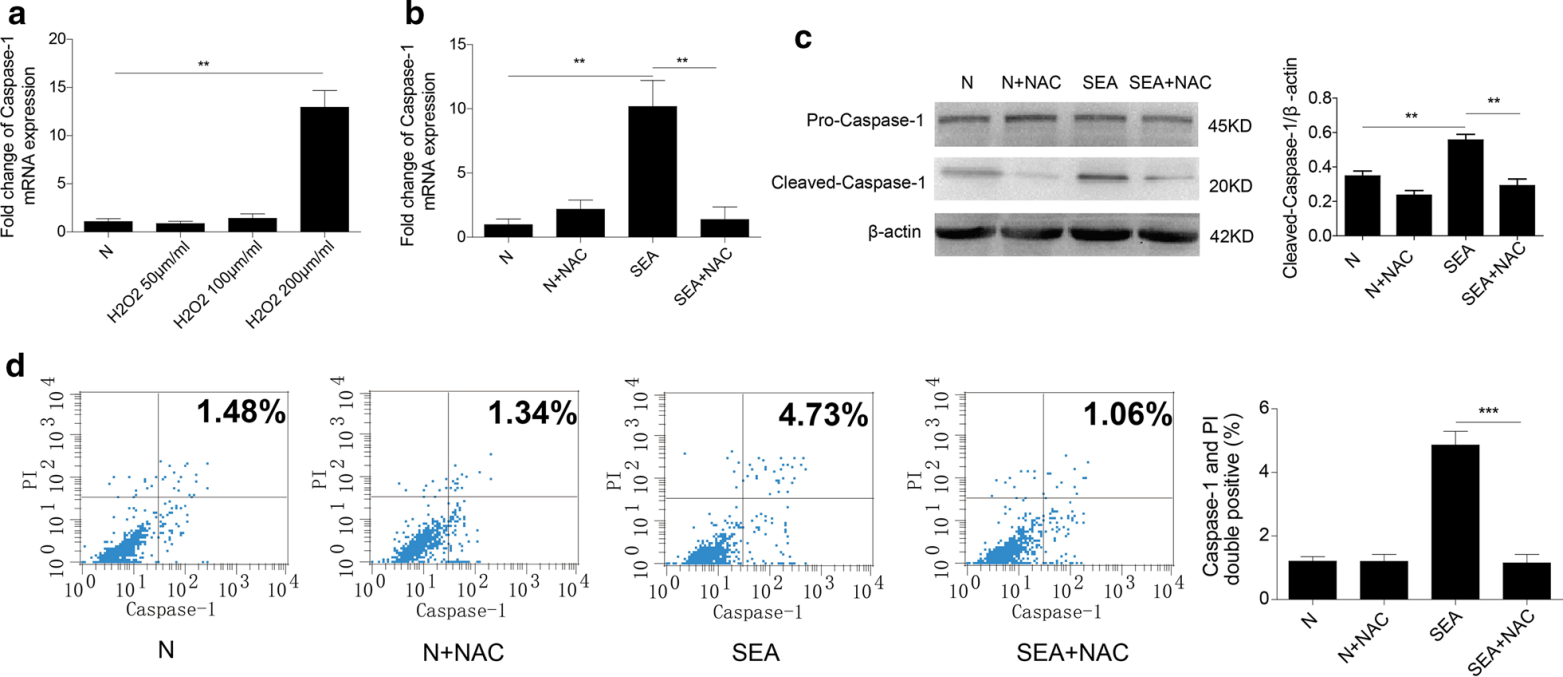
A ROS scavenger mitigated SEA-induced caspase-1 activation and pyroptosis in HSCs. **a** The relative mRNA levels of caspase-1 were determined by qPCR in HSCs after challenge with 50, 100 or 200 μM H_2_O_2_. **b** The effects of the ROS scavenger NAC on the mRNA levels of caspase-1 in HSCs stimulated by SEA were determined by qPCR. **c** The effects of NAC on the protein levels of caspase-1 in HSCs were determined by Western blot analysis and quantitative analysis (*n* = 4). **d** Pyroptotic HSCs labelled with caspase-1 probe and PI were detected by flow cytometry. **P < 0.01, ***P < 0.001

As described above, SEA could dramatically increase intracellular ROS production in HSCs. Therefore, we further investigated the potential role of ROS in SEA-induced caspase-1-dependent pyroptosis. H_2_O_2_ is widely used to induce oxidative stress *in vitro* via the production of intracellular ROS. To explore the mechanism of SEA-induced pyroptosis in HSCs, the effect of H_2_O_2_ on caspase-1 expression in HSCs was determined. Our results showed that 200 μmol/ml H_2_O_2_ caused an apparent increase in caspase-1 expression at the transcriptional level compared to the control (ANOVA: *F*_(3, 12)_ = 9.31, *P* = 0.0022) (Fig. 5a). To determine whether ROS were required for SEA-induced caspase-1 activation in HSCs, cells were pre-treated with the ROS inhibitor NAC and then treated with SEA for 24 h. Our results showed that NAC pre-treatment could inhibit SEA-induced caspase-1 gene expression (ANOVA: *F*_(3, 12)_ = 10.81, *P* = 0.0068) and protein activation (ANOVA: *F*_(3, 12)_ = 13.69, *P* = 0.0017) (Fig. 5b, c). To investigate the role of ROS in SEA-induced pyroptosis, we also analysed the number of caspase-1 and PI double-positive cells in the presence or absence of NAC. As shown in Fig. 5d, the presence of NAC markedly decreased the proportion of double-positive cells after SEA treatment (ANOVA: *F*_(3, 12)_ = 38.99, *P* < 0.0001). Altogether, our results suggested that HSC pyroptosis and caspase-1 activation were dependent on ROS generation.

## Discussion

The present study results showed that SEA could induce the pyroptotic death of HSCs, which was associated with the activation of caspase-1 mediated by ROS in schistosomiasis. To our knowledge, this study is the first to demonstrate pyroptotic death in HSCs induced by SEA, providing a new perspective for understanding the role of SEA-induced functional changes in HSCs in the development of schistosomiasis-associated liver fibrosis.

**Fig. 6:**
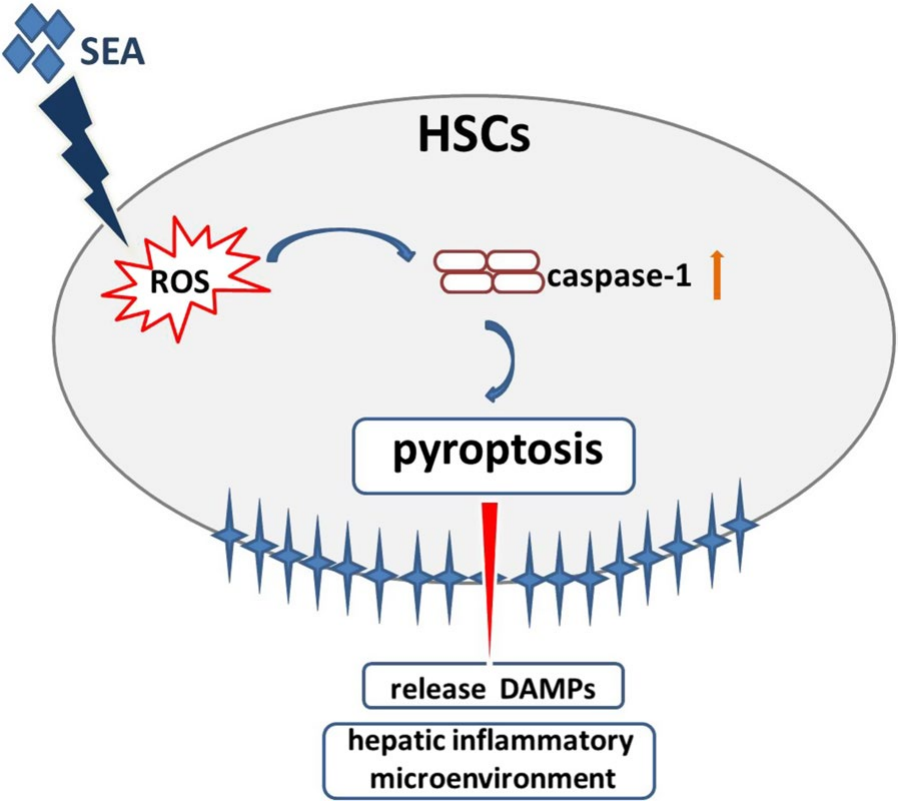
Proposed model of HSCs pyroptosis in SEA-induced schistosomiasis. SEA stimulates HSCs and induces the production of ROS, which leads to the activation of caspase-1. Caspase-1 activation contributes to the pyroptotic cell death and the release of DAMPs which terminally result in the formation of hepatic inflammatory microenvironment, subsequent promoting schistosomiasis. *Abbreviations*: SEA, soluble egg antigen; ROS, reactive oxygen species; HSCs, hepatic stellate cells; DAMPs, damage-associated molecular pattern

Schistosomiasis is a chronic disease that occurs in many regions of the world, and liver fibrosis is its main pathological characteristic of this disease; this fibrosis is caused by the accumulation of parasite eggs in liver tissues, which induces the formation of eosinophilic granulomas [25]. In the present study, we observed that liver granuloma and fibrosis were initiated at 6 weeks p.i. and continued until the chronic liver fibrosis stage at 12 weeks p.i. We also found that both the gene and protein expression of caspase-1, a pro-inflammatory serine protease, was increased during *S. japonicum* infection. All of these results indicated that caspase-1 was involved in schistosomiasis-associated liver fibrosis (Fig. 6).

In response to liver injury, inflammatory mediators and damage-associated molecular patterns (DAMPs) promote HSC activation and differentiation into myofibroblasts in the inflammatory microenvironment, which leads to the deposition of excessive extracellular matrix (ECM) with limited remodelling and inevitably results in fibrosis [26–28]. HSCs are responsible for as much as 80% of total fibrillary collagen in fibrous liver tissues; therefore, the activity of and functional changes in HSCs are considered to be critical factors for the development of both liver inflammation and fibrosis [29, 30]. Given that liver fibrosis often begins at 6 weeks after *S. japonicum* infection in mouse models [31], we isolated mouse HSCs at 6 weeks p.i. and found that caspase-1 expression was increased, indicating that caspase-1 was associated with the activation of HSCs in liver fibrosis during *S. japonicum* infection. Activation of HSCs mediated by causative agents (e.g. SEA) is believed to be a crucial contributor to the development of fibrosis. It has been reported that *S. mansoni* eggs can promote hepatic endothelial cell and fibroblast migration and proliferation [32, 33] as well as collagen synthesis in the liver [34]. In addition, SEA has been shown to upregulate fibrogenesis and inhibit proliferation in primary HSCs [31]. However, the detailed mechanism underlying SEA-induced liver inflammation and injury has not yet been fully clarified.

In response to death-inducing stimuli, healthy cells can initiate a wide variety of molecular pathways that lead to cell death [35]. Cell death is an important pathological characteristic of inflammation and tissue lesions during parasitic infection. Thus far, many forms of cell death, including apoptosis, necrosis, autophagy, pyroptosis, pyronecrosis, necroptosis and oncosis, have been reported [36]. Pyroptosis is mainly mediated by the activation of caspase-1 [8, 37], and the presence of activated caspase-1 is considered to indicate the presence of pyroptosis [38]. Both infectious and non-infectious stimuli can trigger pyroptotic cell death in macrophages, monocytes, dendritic cells, and other cell types, which participates in pathological processes during viral or bacterial infection [39, 40], myocardial ischaemia [41], lung and kidney injury [42, 43], alcoholic hepatitis [44] and cerebral stroke [45]. During pyroptosis, cells exhibit loss of membrane integrity, swelling, lysis, and DNA cleavage. In addition, membrane pore formation leads to secretion of pro-inflammatory cytokines and leakage of intracellular contents or DAMPs [46] such as LDH, which is normally maintained within the cell cytosol. Thus, caspase-1 activation and LDH release assays, combined with dead cell staining, have been used to measure pyroptosis [47, 48]. To the best of our knowledge, the role of pyroptosis in regulating the function of SEA-stimulated HSCs is unknown. In the present study, our results showed that SEA treatment could induce increases in LDH release and decreases in the viability of HSCs. Elevated LDH release indicates the presence of cell damage and lysis in HSCs. Furthermore, our data supported the occurrence of pyroptosis with evidence of double staining for active caspase-1 and nuclei (stained with PI). Taken together, these results indicate that pyroptosis occurred in SEA-stimulated HSCs. Notably, pyroptosis is a pro-inflammatory cell death pattern that can affect the release of pro-inflammatory cytokines [49], and functional abnormalities of HSCs during *S. japonicum* infection are closely related to the presence of a local inflammatory microenvironment. Therefore, in some respects, the pyroptosis-associated release of large amounts of intracellular substances from HSCs into the liver microenvironment may have a clear and direct effect on the development of liver injury caused by *S. japonicum* infection. The detailed effects and importance of pyroptosis in the pathogenesis of schistosomiasis in the liver need further study.

Although the exact mechanism by which caspase-1 causes pyroptosis is unclear, it has been demonstrated that ROS are involved in this pathological mechanism [50, 51]. In the present study, our results showed that ROS generation was elevated in *S. japonicum-*infected mouse livers. Moreover, stimulation with SEA resulted in the accumulation of intracellular ROS in HSCs. H_2_O_2_ is a well-known stimulant that can increase intracellular ROS production in different cells. To elucidate the possible role of ROS in SEA-induced pyroptosis, we treated HSCs with H_2_O_2_ and found that H_2_O_2_ could significantly promote the gene expression of caspase-1 in HSCs. Furthermore, the ROS scavenger NAC not only prevented SEA-induced caspase-1 activation but also inhibited SEA-induced increases in the proportion of cells with double staining for caspase-1 and nuclei (stained with PI). These data indicated that SEA-induced ROS production is an important factor in the induction of pyroptosis in HSCs.

## Conclusions

In the present study, we demonstrated, for the first time, that the activation of caspase-1-associated pyroptosis is mediated by SEA in HSCs. Moreover, ROS generation is involved in SEA-induced caspase-1 activation and pyroptosis, indicating that SEA-induced pyroptotic cell death in HSCs may be a key event in early granuloma formation and may play a vital role in the liver pathogenesis of *S. japonicum* infection. Our data provide novel insights into SEA-induced HSC injury and inflammation, which could deepen our understanding of the pathogenic mechanisms associated with HSC dysfunction in schistosomiasis-associated liver fibrosis and may provide novel drug targets for the treatment of fibrogenesis in this disease.

## Supplementary information

**Additional file 1: Figure S1.** HSCs were cultured in 96-well plates and treated with SEA (50 μg/ml) in the presence or absence of caspase-1 inhibitor belnacasan (VX-765, 20 μM, Selleck, NO. S2228). Cell viability was determined by the method of CCK-8. Graphs represent means ± SD of data from three independent biological replicates. Asterisks indicate statistical significance between the different indicated groups (*P < 0.05, ***P < 0.0001).

**Additional file 2: Figure S2.** HSCs were cultured in 96-well plates and treated with SEA 50 μg/ml in the presence or absence of caspase-1 inhibitor belnacasan VX-765, 20 μM. The supernatant was collected and the release of LDH was measured using LDH detection kit. Graphs represent means ± SD of data from three independent biological replicates. Asterisks indicate statistical significance between the different groups as indicated *P < 0.05.

## Data Availability

All data generated or analyzed in this study are included in this article.

## Abbreviations

SEA: soluble egg antigen
HSCs: hepatic stellate cells
LDH: lactate dehydrogenase
ROS: reactive oxygen species
LSCM: laser scanning confocal microscope
CCK-8: Cell Counting Kit-8
PI: propidium iodide
DAMPs: damage-associated molecular pattern
α-SMA: α-smooth muscle actin
TGF-β1: transforming growth factor beta-1
