# Supplementary figures and images for: Soluble egg antigen of Schistosoma japonicum induces pyroptosis in hepatic stellate cells by modulating ROS production
Source: Parasit Vectors. 2019 Oct 14;12:475. doi: 10.1186/s13071-019-3729-8 (PMC6791022; doi:10.1186/s13071-019-3729-8)

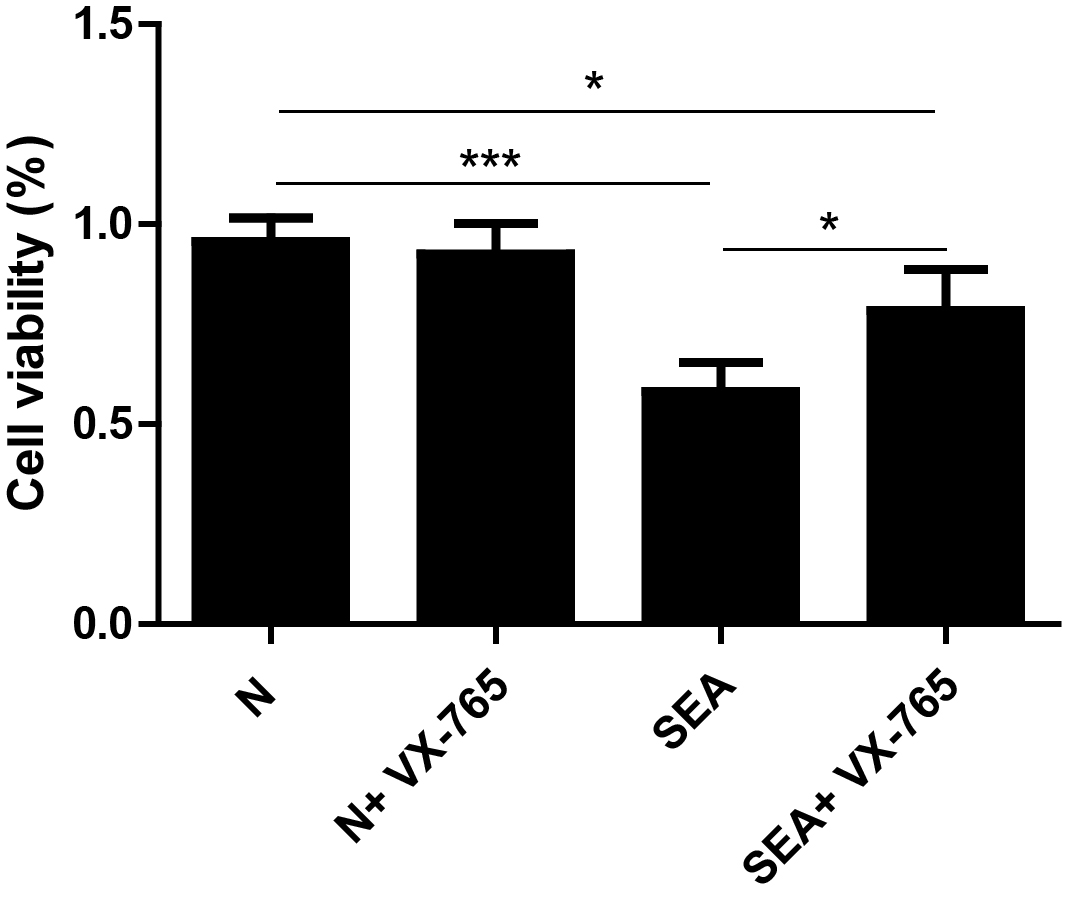

Supplement: Supplementary file 1 — Additional file 1: Figure S1. HSCs were cultured in 96-well plates and treated with SEA (50 μg/ml) in the presence or absence of caspase-1 inhibitor belnacasan (VX-765, 20 μM, Selleck, NO. S2228). Cell viability was determined by the method of CCK-8. Graphs represent means ± SD of data from three independent biological replicates. Asterisks indicate statistical significance between the different indicated groups (*P < 0.05, ***P < 0.0001). [file 13071_2019_3729_MOESM1_ESM.jpg]

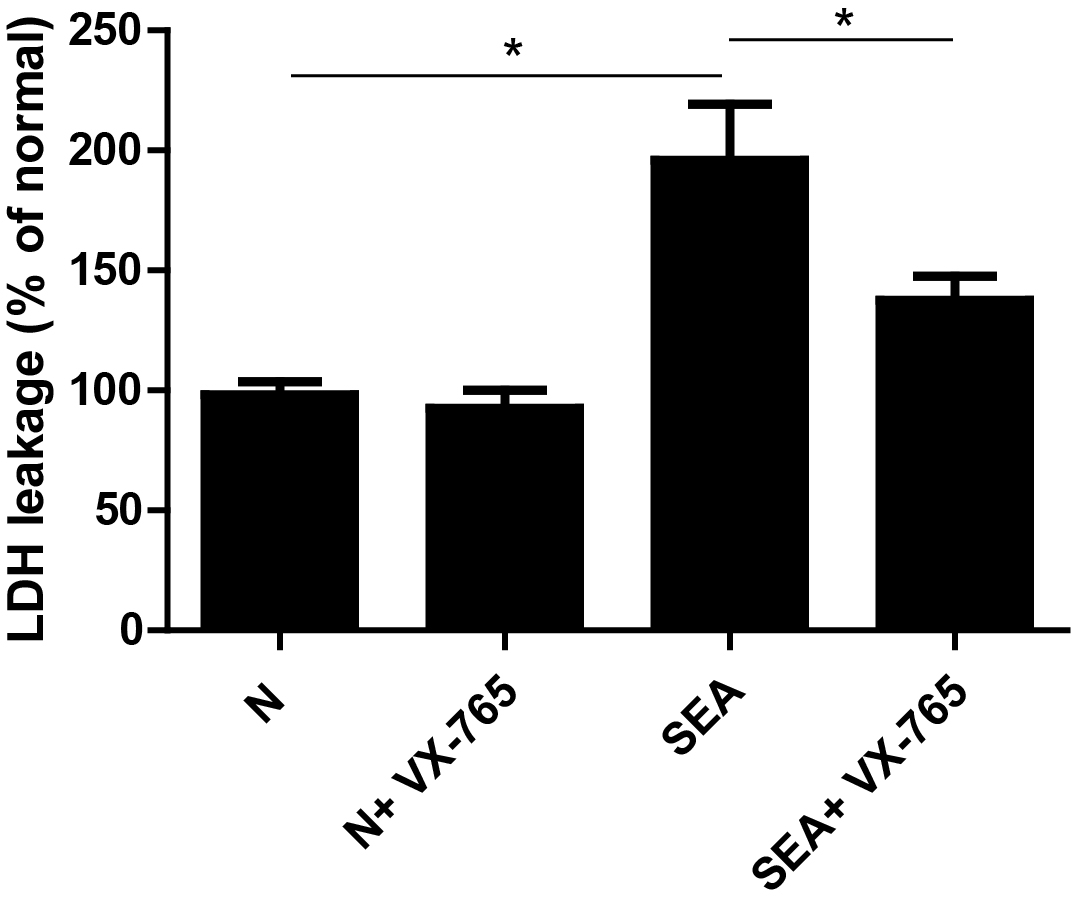

Supplement: Supplementary file 2 — Additional file 2: Figure S2. HSCs were cultured in 96-well plates and treated with SEA 50 μg/ml in the presence or absence of caspase-1 inhibitor belnacasan VX-765, 20 μM. The supernatant was collected and the release of LDH was measured using LDH detection kit. Graphs represent means ± SD of data from three independent biological replicates. Asterisks indicate statistical significance between the different groups as indicated *P < 0.05. [file 13071_2019_3729_MOESM2_ESM.jpg]
